# Correlation of umbilical cord blood haematopoietic stem and progenitor cell levels with birth weight: implications for a prenatal influence on cancer risk

**DOI:** 10.1038/sj.bjc.6604183

**Published:** 2008-02-05

**Authors:** W C Strohsnitter, T M Savarese, H P Low, D P Chelmow, P Lagiou, M Lambe, K Edmiston, Q Liu, I Baik, K L Noller, H-O Adami, D Trichopoulos, C-C Hsieh

**Affiliations:** 1 Department of Obstetrics and Gynecology, Tufts-New England Medical Center, Boston, MA, USA; 2 Department of Cancer Biology and Cancer Center, University of Massachusetts Medical School, Worcester, MA, USA; 3 Department of Neurology, University of Massachusetts Medical School, Worcester, MA, USA; 4 Department of Hygiene and Epidemiology, School of Medicine, Athens University, Athens, Greece; 5 Department of Medical Epidemiology and Biostatistics, Karolinska Institutet, Stockholm, Sweden; 6 Division of Hematology/Oncology, Department of Medicine, UMass Medical School and UMass Memorial Health Care, Worcester, MA, USA; 7 Department of Epidemiology, Harvard School of Public Health, Boston, MA, USA

**Keywords:** birth weight, cancer risk, prenatal exposure, stem cell

## Abstract

We examined the relation with birth weight and umbilical cord blood concentrations of haematopoietic stem and progenitor populations in 288 singleton infants. Across the whole range of birth weight, there was a positive relation between birth weight and CD34^+^CD38^−^ cells, with each 500 g increase in birth weight being associated with a 15.5% higher (95% confidence interval: 1.6–31.3%) cell concentration. CD34^+^ and CD34^+^c-*kit*^+^ cells had J-shaped relations and CFU-GM cells had a U-shaped relation with birth weight. Among newborns with ⩾3000 g birth weights, concentrations of these cells increased with birth weight, while those below 3000 g had higher stem cell concentrations than the reference category of 3000–3499 g. Adjustment for cord blood plasma insulin-like growth factor-1 levels weakened the stem and progenitor cell–birth weight associations. The positive associations between birth weight and stem cell measurements for term newborns with a normal-to-high birth weight support the stem cell burden hypothesis of cancer risk.

The *in utero* environment and perinatal factors may influence cancer risk of the offspring later in life (Trichopoulos, 1990). One parameter that reflects *in utero*/perinatal influences, that is birth weight, has been positively correlated with subsequent risk of childhood cancer (Schüz and Forman, 2007) and, in adults, breast (Michels and Xue, 2006), prostate (Eriksson *et al*, 2007) and colorectal cancers (Nilsen *et al*, 2005) and, indeed, overall cancer risk (Ahlgren *et al*, 2007). Mechanistically, a ‘stem cell burden’ theory (Adami *et al*, 1995) has been proposed to account for the positive relationship between birth weight and the risk of certain cancers, especially that of the breast. By this hypothesis, the levels of *in utero*/perinatal mitogens and other factors determine the size of the stem cell pools in the developing fetus; elevated tissue stem cell numbers drive the formation of larger organs and hence might be associated with larger birth weights. The greater the stem cell pool size, however, the greater the chance that one of the stem cells will be mutated by a carcinogen, or undergo a DNA replicative error, initiating oncogenic transformation. Hence, individuals with high birth weights might be at greater lifetime cancer risk (Trichopoulos *et al*, 2005).

The stem cell burden theory predicts (1) that the *in utero* levels of particular mitogens should correlate positively with stem cell population levels and (2) that the stem cell levels should correlate positively with birth weight. In a previous study, we demonstrated that the umbilical cord blood concentrations of various haematopoietic stem and progenitor populations correlated with cord blood plasma levels of particular mitogens, especially insulin-like growth factor-1 (IGF-1) (Savarese *et al*, 2007). Here, we determine whether or not these measurable haematopoietic stem cell/progenitor values, serving as surrogates of overall stem cell potential, are positively associated with birth weight.

## MATERIALS AND METHODS

The umbilical cord blood study protocol was approved by the institutional review boards of the American Red Cross, the University of Massachusetts Medical School, the University of Massachusetts/Memorial Health Care System, St. Vincent's Hospital and Tufts-New England Medical Center (T-NEMC). Consenting study subjects were recruited from one of two sources: (1) participants in the Worcester, MA-based American Red Cross cord blood program (ACBP), in which a haematopoietic stem cells from umbilical cord blood were collected for possible transplantation, from August 2002 to June 2003, and (2) pregnant women delivering at T-NEMC from October 2004 to April 2006. All the cord blood samples were from full-term (gestational age ⩾37 weeks) singleton infants. The processing of samples, which includes the determination of cord blood volumes, the determination of initial levels of total nucleated cells (TNC) and mononuclear cells (MNC) before centrifugations or manipulation, the quantitation of haematopoietic stem/progenitor cell populations and the determination of cord blood plasma hormone levels, have been described (Savarese *et al*, 2007). The haematopoietic stem and progenitor populations that were quantitated (1) CD34^+^ cells, a heterogeneous population of early multipotent stem and progenitor cells, committed progenitors and differentiating cells (Xiao and Dooley, 2000); (2) CD34^+^CD38^−^ cells, which represent more primitive stem cells depleted of lineage-committed precursors (Xiao and Dooley, 2000); (3) CD34^+^c-*kit*^+^ cells, which also represent a more primitive stem cell population that has relatively high cloning efficiencies in semisolid culture (Sharkey *et al*, 1994; Mayani and Lansdorp, 1998); and (4) granulocyte–macrophage colony forming units (CFU-GM), a functional measure of the number of proliferative granulocyte/macrophage-committed haematopoietic precursor cells (Abboud *et al*, 1992; Hoffbrand *et al*, 2001).

Birth weight was first studied as a categorical variable (<3000, 3000–3499, 3500–3999 and ⩾4000 g). Geometric means for the stem cell measurements were estimated within the indicated categories of birth weight. Multivariate linear regression was used to examine the association between natural log-transformed measures of stem cell potential (dependent variable) and birth weight (independent variable, using 3000–3499 g as the reference), adjusting for maternal and neonatal characteristics (mother's age, race of parents, number of live births, gestation duration, baby's gender, delivery time and study site). To assess whether there was an underlying linear trend, birth weight was next analyzed as a continuous variable across the whole range of birth weight values with the effect estimates expressed for each 500 g increase in birth weight. The fitted coefficients from the regression analyses were exponentiated to obtain the estimated proportional change in birth weight associated with each independent variable. Statistical significance was set at 0.05 (two-sided). Levels of IGF-1, which had the strongest association with levels of stem cells among the hormones and growth factors examined in a previous analysis (Savarese *et al*, 2007), were further adjusted to explore its influence on the association between birth weight and stem cell measurements.

## RESULTS

The characteristics of the study subjects are shown in Table 1. Subjects from the ACBP and T-NEMC patient groups had similar age and gestation duration. Parental ethnicity was more varied in the T-NEMC samples, while the ACBP subjects had higher parities, more male newborns and lower birth weights.

The associations were analysed in multivariate analysis adjusting for maternal age, parental race, parity, gestation duration, gender, delivery time and study site (Table 2, upper panel). There was a J-shaped association between birth weight categories and concentrations of TNC (lymphocytes, monocytes and granulocytes), as well as a J-shaped relation with MNC (lymphocytes and monocytes), both including more differentiated cells. Among the stem cell populations, there was a positive association with CD34^+^CD38^−^ cells across the whole range of birth weight categories, with each 500 g increase being associated with 15.5% higher levels of this cell population (95% confidence interval: 1.6, 31.3%). A J-shaped relation was observed for the CD34^+^ and CD34^+^c-*kit*^+^ cells: for birth weights of 3000 g or greater, stem cell concentrations increased with birth weight, while the lowest category of <3000 g had higher levels than the category of 3000–3499 g. For CFU-GM, an approximate U-shaped relation was observed, with the lowest birth weight category having the highest levels of this cell population (Table 2, upper panel).

Adjusting for cord blood plasma levels of IGF-1 in the multivariate analysis weakens the association (Table 2, lower panel). The association with CD34^+^CD38^−^ remained positive but was no longer statistically significant: each 500 g increase in birth weight was associated with a 7.9% increase in this cell sub-population (95% confidence interval: −6.2, 24.0%). The lowest weight category continued to have the highest CFU-GM cells after adjusting for IGF-1 levels.

We conducted further analyses adjusting for other hormones and in samples from different ethnic groups. Adjusting for estriol or insulin-like growth factor binding protein-3, which had statistically significant but weaker associations with cord blood levels of stem cells (Savarese *et al*, 2007), in place of IGF-1, had much less effect on the association with birth weight (data not shown). A linear relation between stem cell measurements and birth weight was observed for newborns whose parents were Caucasian, but did not have a consistent shape among samples in the mixed non-Caucasian group (Table 3).

## DISCUSSION

The stem cell burden hypothesis has been invoked as an explanation for the positive link between birth weight and risk for both childhood and adult cancers (Adami *et al*, 1995). This hypothesis proposes that *in utero* environments that promote expansion of stem cell pools result in infants with high birth weights; the larger the stem cell pool, the greater the risk that one of these stem cells will undergo malignant transformation. In support of the first tenet of this hypothesis, we have demonstrated that the concentrations of stem and progenitor cell populations in umbilical cord blood, serving as surrogates for overall stem cell potential, correlate with cord blood plasma levels of certain mitogens, notably IGF-1 (Savarese *et al*, 2007). The hypothesis also predicts that newborns with high birth weights should have elevated stem cell populations. Our findings indicate that there is a positive association between birth weight and haematopoietic stem cell measurements in the cord blood samples among newborns with normal-to-high (⩾3000 g) birth weights. This association is strongest with CD34^+^CD38^−^ cells, a relatively primitive haematopoietic stem cell population. These data are in line with previous studies, which showed a positive relationship between cord blood CD34^+^ or CFU-GM levels and birth weight (Shlebak *et al*, 1998; Ballen *et al*, 2001; Aroviita *et al*, 2004).

However, in our study, newborns in the lowest birth weight category (<3000 g) can have higher levels of stem/progenitor cell measurements than those with 3000–3499 g birth weight resulting in a J- or U-shaped relation between stem cell levels and birth weight. This finding is intriguing, as J- or U-shaped relationships have often been observed in childhood cancers (Schüz and Forman, 2007) and neurological (Schüz *et al*, 2001; Von Behren and Reynolds, 2003), prostate (Eriksson *et al*, 2007), colorectal (Nilsen *et al*, 2005) and early-onset breast cancers (Sanderson *et al*, 1996; Innes *et al*, 2000; Mellemkjaer *et al*, 2003).

The possible elevated levels of stem cells at low birth weight warrant further investigations. Using birth weight to define small-for-gestation for full-term healthy infants, a majority (>86%) of such infants undergo accelerated growth during the first 6–12 months after birth and attain normal height later in life (Karlberg and Albertsson-Wikland, 1995). Premature infants (i.e., those born before the 33rd gestational week, generally with low birth weights) have been shown to have elevated foetal and cord blood CD34^+^ and CD34^+^CD38^−^ levels relative to full-term infants (most with a normal birth weight) (Shields and Andrews, 1998; Wyrsch *et al*, 1999). Relevant to this phenomenon, it has been reported that premature female newborns have an increased risk for breast cancer later in life (Ekbom *et al*, 2000). It is not known whether premature or small newborns harbour elevated stem/progenitor cell populations, because these populations have not had enough developmental time to undergo a normal course of differentiation, or if such infants build up a stem cell reserve for growth compensation during the postnatal period. In any case, our J-shaped relation observed between birth weight and stem cell measurements requires further confirmation as ethnicity-specific results showed a linear relation in the considerably larger group of Caucasian cord blood samples.

Finally, we examined cord blood plasma IGF-1 levels in relation to stem cell levels and birth weight. Insulin-like growth factor-1 had a positive association with stem cell measurements in our samples (Savarese *et al*, 2007) and was strongly associated with birth weight (geometric means were 41.18, 57.86, 78.45 and 102.77 ng ml^−1^, respectively, for the four categories of birth weight in Table 2 and in the literature (Bennett *et al*, 1983; Fant *et al*, 1993; Reece *et al*, 1994)). The growth hormone/IGF-1 axis has been suggested to serve as a master developmental regulator, coordinating stem cells in multiple organs (Ginestier and Wicha, 2007). When we controlled for cord plasma IGF-1 levels, the associations were considerably weakened. This would be expected if IGF-1 regulates stem cell potential and this is, in turn, a determinant of birth weight. To determine whether birth weight is an accurate reflection of stem cell potential, the focus should be on the results without adjusting for IGF-1.

We conclude that there is a J- or U-shaped positive association between birth weight and stem cell measurements, linear among term newborns with normal-to-high birth weight and stronger with the more primitive stem cell sub-population. The nonlinear relationship, however, suggests that birth weight is not an unequivocal indicator of stem cell potential in the context of a prenatal origin of cancer risk. Quantitation of stem cell pools might prove to be a more accurate predictor of cancer risk than birth weight *per se*, in accordance with the stem cell burden hypothesis.

## Figures and Tables

**Table 1:** Characteristics of the study subjects by study site

	**ACBP (*n*=39)**	**T-NEMC (*n*=249)**
**Characteristics**	**Mean±s.d. or *N* (%)**	**Mean±s.d. or *N* (%)**
Mother's age (years)	29.7±5.1	30.1±5.6
		
*Parity*		
1	14 (35.9)	104 (45.0)
2	12 (30.8)	64 (27.7)
3	7 (18.0)	39 (16.9)
4 or more	6 (15.4)	24 (10.4)
		
*Race/ethnicity of mother and biological father*		
Both Caucasian	36 (92.3)	111 (52.4)
Both African-American	1 (2.6)	19 (9.0)
Both Asian	0 (0.0)	39 (18.4)
Both Hispanic	0 (0.0)	14 (6.6)
Mixed	2 (5.1)	29 (13.7)
		
Gestation duration (weeks)	39.6±1.4	39.6±1.2
		
*Newborn gender*		
Male	21 (55.3)	113 (50.0)
Female	17 (44.7)	113 (50.0)
		
*Birth weight (g)*	3313.1±450.6	3416.4±428.2
<3000	11 (28.2)	40 (16.1)
3000–3499	15 (38.5)	110 (44.2)
3500–3999	11 (28.2)	77 (30.9)
⩾4000	2 (5.1)	22 (8.8)

Abbreviations: ACBP=American Red Cross Cord Blood Program; T-NEMC=Tufts-New England Medical Center.

Data on some variables were unavailable for subjects with missing values.

**Table 2:** Multiple linear regression analysis for the association between measurements of haematopoietic stem cell populations and birth weight

		**Birth weight (g) in categories**				**Birth weight per 500 g**
**Analytic model**	**Cell measurements**	**<3000 % Difference (95% CI)**	**3000–3499 Reference (geometric mean^a^)**	**3500–3999 % Difference (95% CI)**	**⩾4000 % Difference (95% CI)**	**% Difference (95% CI)**
Adjusted for core covariates^b^	TNC	2.1 (−8.5, 14.0)	0.0 (15.00)	7.8 (−1.7, 18.3)	8.4 (−8.1, 27.8)	3.0 (−2.5, 8.9)
	MNC	5.7 (−6.4, 19.3)	0.0 (6.91)	6.5 (−3.9, 18.0)	12.5 (−6.2, 34.9)	2.1 (−3.9, 8.4)
	CD34^+c^	2.4 (−19.2, 29.7)	0.0 (7.04)	12.5 (−8.1, 37.7)	20.8 (−15.5, 72.8)	9.5 (−2.6, 23.2)
	CD34^+^CD38^−c^	−1.1 (−23.6, 28.1)	0.0 (3.11)	19.1 (−4.3, 48.1)	**47.9 (0.4, 117.8)**	**15.5 (1.6, 31.3)**
	CD34^+^c-*kit*^+d^	13.7 (−14.0, 50.4)	0.0 (5.80)	18.9 (−5.5, 49.6)	34.2 (−10.4, 101.1)	10.0 (−4.0, 26.1)
	CFU-GM^e^	**37.0 (2.1, 83.8)**	0.0 (4.04)	29.0 (−0.1, 66.6)	31.4 (−16.2, 106.1)	6.1 (−8.4, 23.0)
						
Adjusted for core covariates and IGF-1	TNC	0.6 (−10.0, 12.5)	0.0 (15.00)	9.7 (−0.4, 20.8)	12.3 (−5.5, 33.4)	4.5 (−1.5, 11.0)
	MNC	4.0 (−8.0, 17.7)	0.0 (6.91)	8.5 (−2.5, 20.6)	16.8 (−3.5, 41.4)	3.7 (−2.9, 10.8)
	CD34^+c^	11.0 (−12.3, 40.6)	0.0 (7.04)	2.5 (−16.5, 25.8)	−0.3 (−31.0, 44.0)	0.6 (−11.4, 14.2)
	CD34^+^CD38^−c^	5.2 (−19.0, 36.6)	0.0 (3.11)	10.9 (−11.5, 38.8)	27.6 (−14.7, 90.9)	7.9 (−6.2, 24.0)
	CD34^+^c-*kit*^+d^	22.8 (−6.9, 62.1)	0.0 (5.80)	7.0 (−15.3, 35.2)	10.5 (−26.9, 66.9)	0.7 (−12.9, 16.4)
	CFU-GM^e^	**45.6 (8.6, 95.1)**	0.0 (4.04)	15.5 (−11.6, 50.9)	11.5 (−29.8, 77.1)	−1.7 (−16.2, 15.3)

a Unadjusted geometric means. TNC, initial total nucleated cells × 10^6^ per ml; MNC, initial mononuclear cells × 10^6^ per ml; the unit for the stem cell populations (CD34^+^, CD34^+^CD38^−^, CD34^+^c-*kit*^+^, and CFU-GM) was per 1000 MNC.

b Core covariates included mother's age, race of parents (both Caucasian or not), parity, gestation duration, gender of baby (male or female), delivery time (night or day) and study site (ACBP or T-NEMC).

c *n*=233 with complete information on all the covariates.

d Determined only in the T-NEMC-derived samples.

e Data from the T-NEMC-derived samples on which this assay was conducted. Statistical significance of P<0.05 are given in bold.

**Table 3:** Ethnic-specific, core covariate-adjusted^a^ multiple linear regression analysis for the association between measurements of haematopoietic stem cell populations and birth weight

		**Birth weight (g) in categories**				**Birth weight per 500 g**
**Ethnicity**	**Stem cell measurements**	**<3000 % Difference (95% CI)**	**3000–3499 Reference (geometric mean^b^)**	**3500–3999 % Difference (95% CI)**	**⩾4000 % Difference (95% CI)**	**% Difference (95% CI)**
Both parents Caucasian	CD34^+^	−13.9 (−37.2, 18.0)	0.0 (7.73)	8.3 (−15.2, 38.3)	37.5 (−11.2, 113.1)	**16.6 (0.9, 34.7)**
	CD34^+^CD38^−^	−18.0 (−43.0, 17.9)	0.0 (3.41)	14.3 (−13.7, 51.4)	**80.5 (9.1, 198.7)**	**23.8 (4.7, 46.4)**
	CD34^+^c-*kit*^+^	−11.5 (−40.8, 32.3)	0.0 (6.44)	18.4 (−11.0, 57.5)	63.4 (−1.0, 169.5)	**22.9 (3.6, 45.8)**
	CFU-GM	19.3 (−25.9, 92.8)	0.0 (4.01)	36.9 (−3.4, 94.1)	40.0 (−25.3, 162.5)	19.7 (−3.3, 48.2)
						
Either parent non-Caucasian	CD34^+^	28.4(−12.1, 87.6)	0.0 (6.47)	19.9 (−16.6, 72.3)	−0.6 (−46.6, 85.3)	0.5 (−18.1, 23.5)
	CD34^+^CD38^−^	23.2 (−16.2, 81.2)	0.0 (2.84)	28.2 (−10.8, 84.4)	13.5 (−39.1, 111.7)	6.3 (−13.8, 31.0)
	CD34^+^c-*kit*^+^	42.1 (−6.2, 115.5)	0.0 (5.39)	22.2 (−17.2, 80.4)	9.2 (−43.9, 112.6)	−0.4 (−20.4, 24.7)
	CFU-GM	**68.4 (14.0, 148.6)**	0.0 (4.08)	21.0 (−18.1, 78.6)	24.3 (−37.5, 147.1)	−7.3 (−26.0, 16.1)

a Core covariates included mother's age, parity, gestation duration, gender of baby (male or female), delivery time (night or day) and study site (ACBP or T-NEMC).

b Unadjusted geometric means. The unit for the stem cell measurements was per 1000 MNC. Statistical significance of P<0.05 are given in bold.
